# Impact of Various Extraction Technologies on Protein and Chlorophyll Yield from Stinging Nettle

**DOI:** 10.3390/foods13203318

**Published:** 2024-10-18

**Authors:** Simon Dirr, Özlem Özmutlu Karslioglu

**Affiliations:** 1 Faculty of Food Technology and Horticulture, University of Applied Sciences Weihenstephan-Triesdorf, Am Hofgarten 4, 85354 Freising, Germany; oezlem.oezmutlu@hswt.de; 2 Institute of Food Technology, University of Applied Sciences Weihenstephan-Triesdorf, Am Staudengarten 11, 85354 Freising, Germany

**Keywords:** *Urtica dioica*, color intensity, pulsed electric fields, high-pressure homogenization, protein extraction

## Abstract

Stinging nettle (*Urtica dioica* L.) has gained attention as a sustainable protein source due to its rich bioactive compound profile and medicinal properties, but research on optimizing its protein extraction remains limited. This research explores various cell disruption methods, including pulsed electric fields and high-pressure homogenization, combined with extraction techniques like isoelectric precipitation, ultrafiltration, and salting-out, to enhance protein yield and assess its impact on chlorophyll content. The findings indicate that high-pressure homogenization combined with isoelectric precipitation achieved the highest protein yield of 11.60%, while pulsed electric fields with ultrafiltration significantly reduced chlorophyll content from 4781.41 µg/g in raw leaves to 15.07 µg/g in the processed sample. Additionally, the findings suggest that innovative extraction technologies can improve the efficiency and sustainability of protein isolation from stinging nettle, offering a valuable addition to the repertoire of alternative protein sources. These advancements could pave the way for broader applications of stinging nettle in food fortification and functional ingredient development.

## 1. Introduction

In covering the worldwide protein needs through alternative and sustainable resources, green leaf proteins have been addressed in research in the last decade. As one of these potential sources, stinging nettle has also been studied for its properties as well as to find out optimized extraction processes. *Urtica dioica* L., commonly known as stinging nettle, is a perennial herbaceous plant belonging to the family *Urticaceae*. It is distributed in temperate regions in many parts of the world, including areas in Asia, Europe, North Africa, and North America, up to altitudes of 1800 m [1]. Stinging nettle is commonly linked with its diuretic effects and its role in treating urinary tract infections. Moreover, it is recognized as possessing antioxidant, anti-inflammatory, hypoglycemic, and hypocholesterolemic properties. These beneficial effects stem from the presence of biologically active compounds in nettle, including phenolic acids (such as protocatechuic, quinic, coumaric, coffee, and ferulic), tannins, pigments, unsaturated fatty acids, sterols, and phytoestrogens [2]. Nettle leaves are abundant in chlorophyll, carotenoids, vitamins, proteins, fats, carbohydrates, organic acids, minerals, and trace elements. The primary carotenoids found in these leaves include β-carotene, violaxanthin, xanthophylls, zeaxanthin, luteoxanthin, and lutein epoxide [3]. Stinging nettle contains both water-soluble vitamins, significant amounts of vitamin C and vitamin B (B1, B2, B3, B6, B9), and fat-soluble vitamins A, D, E, K, pro-vitamin A (β-carotene), and vitamin E (α-tocopherol). The nutritional composition of fresh leaves of stinging nettle is as follows: water content is between 65% and 90%, protein content varies from 4.3% to 8.9%, carbohydrates range from 7.1% to 16.5%, ash content is between 3.4% and 18.9%, lipids range from 0.7% to 2%, and fibers vary from 3.6% to 5.3% according to Said et al., 2015 [4].

Although there is a variation in protein content depending on the locality of the plants, there are several reports on protein content. In a study where samples were taken from the six regions of the Republic of Macedonia, protein content ranged from 16.08% to 26.89% [5]; in another study where commercialized samples from Germany were used, protein determination analysis delivered an amount of crude protein of 32.3%.

Due to its valuable composition, stinging nettle has been used in various food applications for fortification purposes. It has been used as a coagulant to inoculate milk to make fresh cheese, where it has been introduced as an ingredient in gastronomy to increase the sensory appeal of vegetarian cheeses and yogurt [6]. To boost the effectiveness of the bioactive compounds in nettle, efforts have been made to encapsulate its extracts in nanoparticles that could be incorporated into various food products. Nettle is rich in chlorophyll and can be used as a natural coloring agent alongside vegetable powders and concentrates [7].

So far, the extraction of bioactive compounds for stinging nettle has been heavily researched. Several studies suggest that various extraction techniques, including ultrasound-assisted, microwave-assisted, subcritical water, and high-pressure-assisted extraction, have been explored for stinging nettle, focusing on the identification of biologically active compounds and potential therapeutic applications for inflammatory diseases [8]. Research on protein extraction is rather limited and has been focused on using innovative technologies to reduce solvent or usage of excess chemicals and to increase effectivity. Pulsed electric fields (PEF), a nonthermal technology known in food science for increasing cell membrane permeability, have already been applied and optimized for protein extraction.

This study aimed to investigate the effect of different cell disruption methods like PEF and High-Pressure Homogenization (HPH) and extraction technologies such as Isoelectric Precipitation (IEP), Ultrafiltration (UF), and Salting-Out (SO) to increase the protein yield and assess its effect on chlorophyll content.

## 2. Materials and Methods

### 2.1. Materials

Nettle leaves were provided dried, partially fragmented, with irregular dimensions (<3 mm) by Holger Senger Vertrieb von Naturrohstoffen e.K., Dransfeld, Germany. All analyses were conducted with the same batch. The samples were stored at room temperature (20 °C) prior to treatment. The current study was performed with dried leaves to standardize the raw material.

### 2.2. Methods

**Figure 1:**
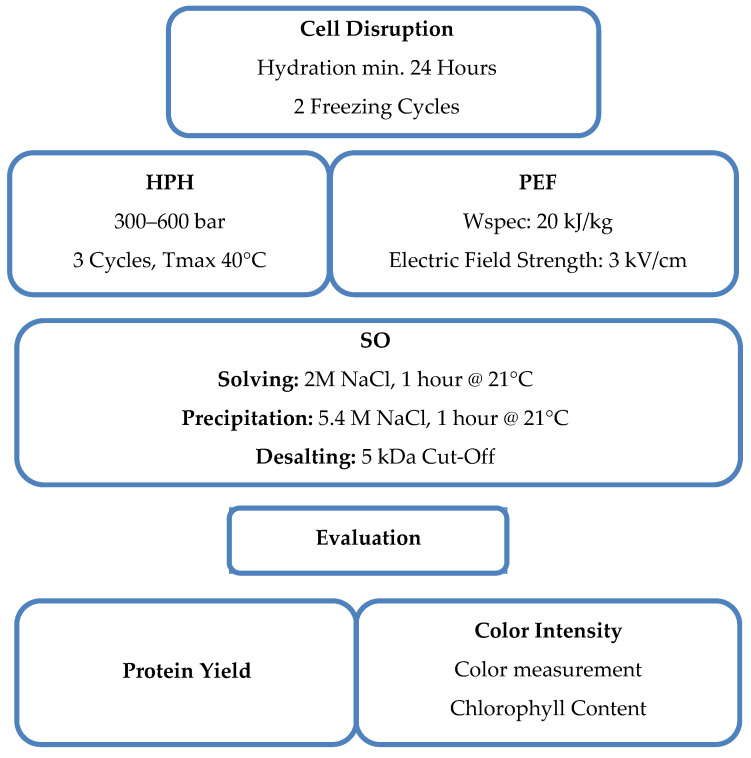
Schematic diagram illustrating the evaluated parameters and the methodology, providing an overview of the study’s content and approach.

A comprehensive understanding of the evaluated parameters and the methodical approach undertaken within this study is graphically expressed by the schematic diagram in Figure 1. This figure serves as a visual summary of the key components and their alignment with the study aim. A detailed explanation of each aspect can be found in the following chapters.

### 2.3. Sample Preparation

Nettle leaves were ground using Thermomix TM6, Vorwerk Deutschland Stiftung & Co. KG, Wuppertal, Germany (Level 10, 1 min), then a ball mill at 400 rpm for 5 min with 20 balls (PM100, Retsch GmbH, Haan, Germany) and sieved using a sieving tower (AS400 control, Retsch GmbH, Haan, Germany) with a final mesh size of 125 µm. The fraction with 125 µm was stored at 21 °C until further use. A 5% (*w*/*w*) dispersion of stinging nettle powder (SN5%) and distilled water was produced using an Ultra Turrax Miccra D-9, MICCRA GmbH, Buggingen, Germany, at 19,000 rpm for 4 min, and frozen at −21 °C until further use. Before conducting the cell disruption methods, the dispersion was thawed at room temperature for 24 h. After the cell disruption, all dispersions were frozen at −21 °C a second time to account for shipping conditions and comparability.

### 2.4. Cell Disruption

#### 2.4.1. High-Pressure Homogenization (HPH)

The thawed extract was homogenized in 3 cycles at 300–600 bars with a two-stage homogenizer (Panda Plus 2000, GEA Niro Soavi, Parma, Italy) to reach a particle size distribution of d90 < 150 µm. During this process, the temperature did not exceed 40 °C. After homogenizing, the extract was frozen at −21 °C until further use similar to the method of Goktayoglu Ece et al., 2023 [9].

#### 2.4.2. Pulsed Electric Field (PEF)

SN5% was shipped to the project partner Elea Technology GmbH, Quakenbrück, Germany. PEF treatment was conducted according to Kronbauer et al., 2023 [10]. The sample was treated in the Elea Advantage System with a specific energy (wspec) of 20 kJ/kg and an electric field strength of 3 kV/cm. High-voltage exponential decay, monopolar pulses with an interval of 0.5 s (2 Hz), and pulse duration of 40 ms were supplied by the PEF system. After the PEF treatment, the sample was heated and maintained at 70 °C for 10 min while being stirred by a PC-MKM 1074, Profi Cook, Kempen, Germany. The heated sample was then rapidly cooled to room temperature using an ice bath. Following this, the sample was frozen at −21 °C and transported for further analysis.

### 2.5. Particle Size Distribution

The distribution of the particle size was analyzed using the S3500 Particle Size Analyzer from Microtrac Retsch GmbH, Haan, Germany, with a tri-laser system based on laser diffraction. The analyzer was equipped with a liquid dispersion module, capable of measuring particle sizes ranging from 0.1 to 1000 µm. The cumulative distribution was characterized by determining the values D10, D50, and D90. The percentage volume fraction of the particles is plotted against the particle size, dx, which represents the diameter where 10%, 50%, or 90% of the total particle volume is smaller than dx.

### 2.6. Protein Solubility

To efficiently extract the proteins from the nettle leaves, a solubility curve was generated. The protein solubility was measured at pH levels ranging from 2 to 12 in increments of 1, and between 3.5 and 4.5 in 0.5 increments, with an accuracy of ±0.1. The samples were prepared 24 h in advance to ensure complete hydration. Therefore, 0.5 g of stinging nettle powder was weighed in a 50 mL centrifuge tube, and 50 mL of distilled water was added. The 1% *w*/*v* dispersion was vortexed for 10 s before placing the tubes in a vertical shaker at 25 °C for 24 h at 130 rpm (KS 3000, IKA-Werke GmbH & Co. KG, Staufen, Germany). The following day, the dispersion was transferred to a beaker, and the pH was adjusted with 0.25 M sodium hydroxide (NaOH) (Merck KGaA, Darmstadt, Germany) and hydrochloric acid (HCl) (Merck KGaA, Darmstadt, Germany) and stirred for 2 h. Then, 2 mL of the pH-adjusted samples were transferred into micro-centrifuge tubes and centrifuged at 2860 RCF for 20 min (MIKRO 200, Hettich GmbH & Co. oHG, Kirchlengern, Germany). The supernatant was then analyzed. This method was performed similarly to Mishyna et al., 2019 just with a wider pH range and more initial time for soaking [11]. To quantify the soluble proteins, the Bradford method was used with bovine serum albumin (BSA) (Thermo Fisher Scientific, Waltham, MA, USA) as a reference [12].

In total, 50 µL of supernatant was transferred to a cuvette, and 1.5 mL of Bradford reagent was added (Thermo Fisher Scientific, Waltham, MA, USA). After incubating for 10 min at room temperature, the absorbance was measured using a spectrophotometer at a wavelength of 595 nm (SPECORD 210 PLUS, Analytik Jena GmbH & Co.KG, Jena, Germany) with its corresponding software (Aspect UV, Version 1.2.3 Analytik Jena GmbH & Co.KG, Jena, Germany). Prior to this, the spectrophotometer was zeroed using the absorbance of distilled water.

To calculate the protein solubility, the following formulas were used:

(1) $$C\left[\frac{µg}{mL}\right]=\frac{A_{595}-t}{m}$$

C = concentration of BSA standard at the absorption of the sample (in µg/mL),

A_595_ = absorbance of the sample at the wavelength of 595 nm [A/cm],

t = intersection of the line with the *y*-axis from the BSA calibration curve (Blank),

m = slope of the line from the calibration curve,

(2) $$PS\left[\%\right]=\frac{C\times DF\times V}{m\times 10^{6}\times \frac{DM}{100}\times \frac{P}{100}}\times 100\%$$

PS = protein solubility in %,

C = concentration of BSA standard at the absorption of the sample in µg/mL,

DF = dilution factor (1:1 = 2, 1:2 = 3),

V = volume of water used to hydrate the sample in mL,

M = weight of the initial powder in g,

10^6^: = conversion factor from grams to µg,

DM = dry matter in % (of the initial powder),

P = protein content in % of the sample based on dry matter.

### 2.7. Extraction

#### 2.7.1. Isoelectric Precipitation (Alkaline Solving)

**Figure 2:**
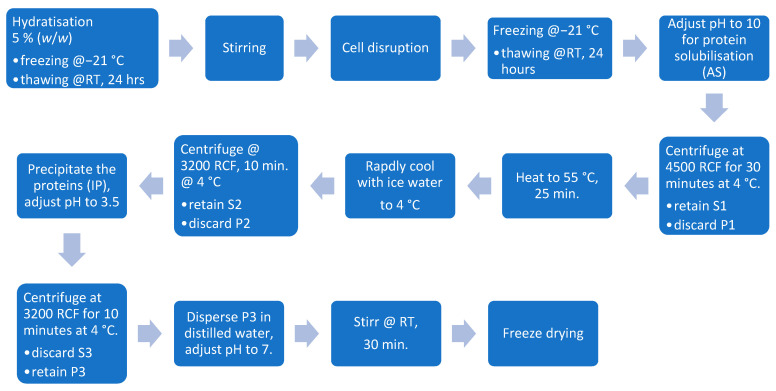
Isoelectric precipitation processing steps.

The method described by Nynäs et al. (2021) was followed with slight modifications for the alkaline solubilization and isoelectric precipitation, including a thermal treatment to decolorize the extracted proteins [13]. After cell disruption, as outlined in Section 2.4.1 and Section 2.4.2, the dispersions were thawed at room temperature for 24 h. The pH was then adjusted to 10, and the dispersion was stirred for 30 min before being centrifuged at 4500 RCF for 30 min at 4 °C (Rotana 460R, Hettich GmbH & Co. oHG, Germany). Following centrifugation, the supernatant (S1) was heated to 55 °C and maintained at that temperature for 30 min using a blender with a heating function (Thermomix TM6, Vorwerk Deutschland Stiftung & Co. KG, Wuppertal, Germany) before being rapidly cooled to 4 °C. The extract was then centrifuged at 3200 RCF for 10 min at 4 °C. The pH of the collected supernatant (S2) was adjusted to 3.5 before another round of centrifugation at 3200 RCF for 10 min at 4 °C. The resulting pellet was diluted in distilled water, stirred for 30 min, and its pH was adjusted to 7 before freeze-drying WAVE FD260, Wave Trockensysteme GmbH, Vienna, Austria. The flow diagram of the process is displayed in Figure 2.

#### 2.7.2. Ultrafiltration

**Figure 3:**
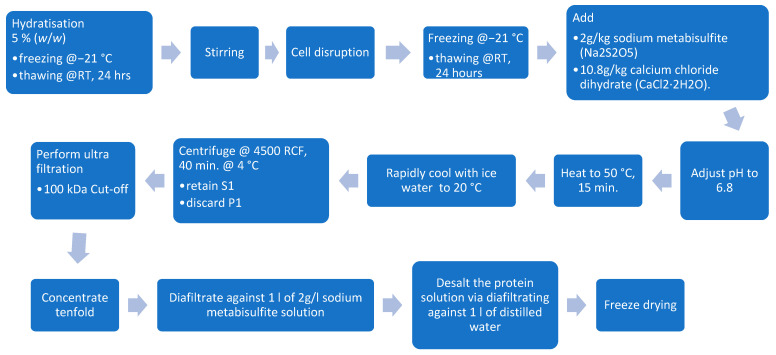
Ultrafiltration processing steps.

Ultrafiltration was used to concentrate the material for protein extraction as well as for the removal of chlorophyll. The method from Martin et al., 2019 was used with slight modifications to the centrifugation settings and raw material selection [14]. After cell disruption, the dispersions were thawed at room temperature for 24 h. After thawing, 2 g/kg sodium metabisulfite (VWR Chemicals Deutschland GmbH, Darmstadt, Germany) and 10.8 g/kg calcium chloride dehydrate (Merck KGaA, Darmstadt, Germany) were added, and the pH was set to 6.8 using 1 M NaOH (Merck KGaA, Darmstadt, Germany) before the dispersion was heated to 50 °C and maintained at this temperature for 15 min (Thermomix TM6, Vorwerk Deutschland Stiftung & Co. KG, Wuppertal, Germany). Following the thermal treatment, the medium was transferred to an ice bath and rapidly cooled to 20 °C before being centrifuged for 40 min at 4500 RCF and 4 °C (Rotana 460R, Hettich GmbH & Co. oHG, Kirchlengern, Germany). The retained supernatant (S1) was then concentrated tenfold using a 100 kDa regenerated cellulose cassette (Hydrosart, Sartorius AG, Göttingen, Germany). The concentrated solution was diafiltrated against 1 L of a solution containing 2 g/L sodium metabisulfite. To desalt the protein-rich solution, it was concentrated tenfold again before diluting 1:1 with distilled water and halving its volume. This dilution step was performed five times before the sample was freeze-dried (WAVE FD260, Wave Trockensysteme GmbH, Vienna, Austria). An overview of the process is shown in Figure 3.

#### 2.7.3. Protein Extraction Utilizing Salting-In and Salting-Out

**Figure 4:**
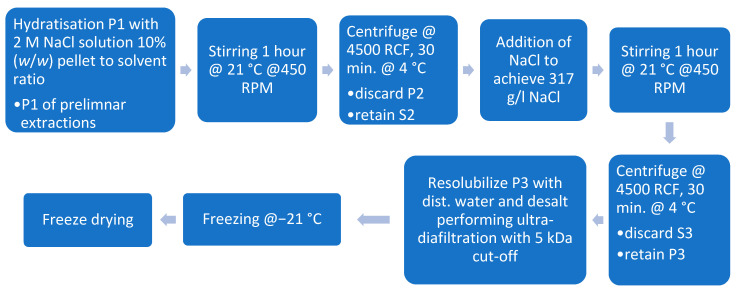
Protein extraction through salting-out with sodium chloride.

To increase protein yield further, a second stage extraction was conducted according to Kusumawati 2020 [15]. Therefore, for the first pellet (P1), as described in Section 2.7.1 and Section 2.7.2, a second extraction cycle was conducted. To enhance protein solubilization, P1 was resolubilized using a 2 M Sodium chloride (NaCl) (Merck KGaA, Darmstadt, Germany) solution at a 1:10 (*w*/*w*) ratio of wet pellet to salt solution. The mixture was incubated for 1 h at 21 °C with continuous stirring at 450 RPM. Following incubation, the dispersion was centrifuged at 4500 RCF for 30 min at 4 °C (Rotana 460R, Hettich GmbH & Co. oHG, Kirchlengern, Germany), and the resulting pellet (P2) was discarded. The salt concentration of the supernatant (S2) was then adjusted by adding crystalline NaCl to achieve a saturated NaCl solution (317 g/L, approximately 5.4 M NaCl) due to the lack of further information on the ionic strength of the proteins. The saturated solution was incubated at 21 °C for 1 h before being centrifuged at 4500 RCF for 30 min at 4 °C. The resulting supernatant (S3) was discarded, and the pellet (P3) was collected. P3 was resolubilized in distilled water and desalted using ultrafiltration with a 5 kDa cut-off membrane (Hydrosart, Sartorius AG, Göttingen, Germany). The protein solution underwent a tenfold concentration step, followed by the addition of an equal volume of distilled water and halving its volume. This process was repeated for five cycles. Finally, the samples were freeze-dried (WAVE FD260, Wave Trockensysteme GmbH, Vienna, Austria) for further analysis. The process can be seen in Figure 4.

### 2.8. Dry Matter

The dry matter of the raw material was determined with a moisture analyzer (KERN DAB 100, KERN & SOHN GmbH, Balingen, Germany) according to the suppliers method [16]. Aluminum weighing trays were placed into the device, and the sample was weighed. For the raw material, the weight was 5 g per measurement, and for the extracted samples, 0.1 g was used due to a limited amount of material. The sample was then heated up to 130 °C and held until a weight equilibrium was reached. The dry matter was then calculated by the device using the following equation:

(3) $$DM[\%]=\frac{m_{sample\;dry}}{m_{ample\;moist}}\times 100\%$$

### 2.9. Total Nitrogen Content

Total nitrogen content was determined using the Kjeldahl method with modifications according to the method of C. Gerhard GmbH & Co. KG, Königswinter, Germany [17]. Therefore, approximately 0.1 g of the sample was weighed in a nitrogen-free weighing boat (Cytiva, Marlborough, MA, USA) and transferred into a Kjeldahl tube. In total, 20 mL of 98% sulfuric acid (Merck KGaA, Darmstadt, Germany) and one Missouri Kjeldahl catalyst tablet (Merck KGaA, Darmstadt, Germany) were added, and the mixture was digested for approximately 3 h at 400 °C in a Kjeldahtherm and Turbosog apparatus (C. Gerhard GmbH & Co. KG, Germany). After digestion and cooling to 60 °C, the samples were steam-distilled with a Vapodest (C. Gerhard GmbH & Co. KG, Königswinter, Germany). This was performed by adding 100 mL of distilled water, and 80 mL of NaOH 33% (*w*/*w*) (Merck KGaA, Darmstadt, Germany) were added to the Kjeldahl tube and distilled for 8 min at 100 °C. The distillate was collected in boric acid 4% (*w*/*w*) and titrated to the initial pH by an auto-titrator (Titroline500, SI Analytics, Mainz, Germany) using 0.1 M HCL (Merck KGaA, Darmstadt, Germany). The total nitrogen content was calculated using Formula (4) and the corresponding protein content using Formula (5), with a standard protein factor of 6.25.

(4) $$N\left[\%\right]=\frac{c_{eq}\times \left(V-V_{BL}\right)\times M\times 100\%}{m_{sample}\times DM}$$

N = nitrogen content in %,

Ceq = normality of the titration solution in mol/L,

V = consumption of titration solution sample in L,

V_BL_ = consumption of titration solution blank value in L,

M = molar mass nitrogen in g/mol,

msample = initial sample weight in g,

DM = dry mater in %.

(5) P[%]=N×F

P = protein content in %,

F = protein factor (6.25).

### 2.10. Protein Yield

Protein yield was determined to evaluate the effectivity of the extraction process and identify further optimization parameters. To calculate the protein yield, Formula (6) was used [13].

(6) $$PY\left[\%\right]=\frac{m_{sample,out}\times P_{out}\times {DM}_{out}}{m_{sample,in}\times P_{in}\times {DM}_{in}}$$

### 2.11. Color Intensity

#### 2.11.1. Color Analysis

Digital capturing and visualization of color differences were performed according to Limbo and Piergiovanni, 2009, by using a DigiEye (VeriVide Ltd., Leicester, UK), and its corresponding software (DigiEye, Version 2.9.0.7, VeriVide Ltd., Leicester, UK) in diffuse mode to remove specular reflection [18]. A standardized light source (CIE D65) was chosen, as most food is viewed in daylight. The angle of observation was set to 10° to simulate a wide field of view. The L* a* b* values were determined, which stand for coordinates in the CIELAB color room. When comparing two different colors, differences in the color coordinates are formed. These are marked with delta ∆. Determination of the total distance ∆E can be calculated via Formula (7). The ∆E indicates the total color difference without direction [18].

(7) $$∆E=\sqrt{{\left(∆L^{*}\right)}^{2}+{\left(∆a^{*}\right)}^{2}+{\left(∆b^{*}\right)}^{2}}$$

#### 2.11.2. Determination of Chlorophyll Through Extraction with Ethanol

The determination of chlorophyll was conducted via the procedure from Ceasar et al., 2018, with slight modifications [19]. Chlorophyll was extracted with ethanol (96%) (VWR Chemicals Deutschland GmbH, Darmstadt, Germany) within two cycles. A total of 20 mg of sample were weighed in a 15 mL falcon tube, and a spatula tip of MgCO_3_ (Merck KGaA, Darmstadt, Germany) and 6 mL of ethanol (96%) were added. As a blank, MgCO_3_ and ethanol were used. For the first extraction, all samples were heated in a water bath at 80 °C until the liquid started to boil and were kept boiling for 5 min. After boiling, the tubes underwent a cooling period of 10 min before being placed on a horizontal shaker for 20 min at 130 rpm. The samples were then centrifuged at 3000 RCF at 21 °C for 10 min (Rotana 460R, Hettich GmbH & Co. oHG, Darmstadt, Germany). The resulting supernatant was transferred into separate tubes, and another 6 mL of ethanol was added to the samples to initiate a second extraction cycle, which followed the same procedure as the initial extraction. Following the second extraction cycle, the supernatants from both extractions were combined, filled up to 12 mL and centrifuged once more. The absorbance of the combined supernatant was then measured spectrophotometrically at wavelengths of 665 nm, 649 nm, and 750 nm (SPECORD 210 PLUS, Analytik Jena GmbH & Co.KG, Jena, Germany). The absorption at 750 nm was included to account for any potential influence of the intrinsic color of the samples. All absorption values were corrected with the blank values before calculating the chlorophyll a and b content using the following Formulas (8)–(10) [19]:

(8) $$Chl\;a\left[µg\right]=13.5275\times (A665-A750)-5.201\times (A649-A750)\times DF\times S$$

(9) $$Chl\;b\left[µg\right]=22.4327\times (A649-A750)-7.0741\times (A664-A750)\times DF\times S$$

Chl a, b = chlorophyll content in µg,

Coefficients (Eλ) Chlorophyll a 649 nm = −5.2007 in $\frac{mg*cm}{l*A}$,

Coefficients (Eλ) Chlorophyll b 649 nm = 22.4327 in $\frac{mg*cm}{l*A}$,

Coefficients (Eλ) Chlorophyll a 665 nm = 13.5275 in $\frac{mg*cm}{l*A}$,

Coefficients (Eλ) Chlorophyll b 665 nm = −7.0741 in $\frac{mg*cm}{l*A}$,

A_x_ = absorbance at specific wavelength in A/cm,

DF = dilution factor,

S = amount of solvent in mL.

The total chlorophyll a + b content was calculated by summing the results obtained from Formulas (8) and (9). The chlorophyll amount in relation to the sample weight is calculated using the following formula:

(10) $$Chl\left(a,b,a+b\right)[\frac{µg}{g}]=\frac{Chl(a,b,a+b)}{m_{sample}\times DM}$$

Chl a, b, a + b = chlorophyll content in µg,

M_sample_ = weight of sample initially weighed in g,

DM = dry mater in %,

The chlorophyll reduction was calculated by using Formula (11).

(11) $${Chl\;a+b}_{red}\left[\frac{µg}{g}\right]={Chl\;a+b}_{Raw}-{Chl\;a+b}_{x}$$

Chl a + b _red_ = reduction in chlorophyll a + b content of the raw material compared to the sample in µg/g,

Chl a + b _red_ = chlorophyll a + b content of the raw material in µg/g,

Chl a + b _x_ = chlorophyll a + b content of the sample in µg/g.

### 2.12. Statistical Analysis

For the statistical analysis, Minitab 21 (Version 10 Minitab Inc., State College, PA, USA) was used with a significance level of α = 5%. The Anderson–Darling test was used to determine the normality of the data. If the *p*-value is less than 0.05, the null hypothesis, which assumes the data follow a normal distribution, is rejected. Conversely, if the *p*-value is 0.05 or greater, there is no sufficient evidence to reject the hypothesis of normality. To assess variance homogeneity, the Levene test is used. The null hypothesis states that the variances are equal. If the *p*-value is less than the α level, the null hypothesis is rejected, indicating that there is no reliable evidence that the variances are equal.

A single-factor ANOVA is used to compare the mean values, where the null hypothesis is that all means are equal. If the *p*-value is smaller than the α level, the null hypothesis is rejected, suggesting that at least one mean value is different. To identify the specific differences, Tukey’s Honest Significant Difference (HSD) post-hoc test was conducted. In the resulting groups, those sharing the same smaller letter have no significant difference.

## 3. Results and Discussion

To facilitate a better understanding of the abbreviations used in the following results within this chapter, the nomenclature for these abbreviations is provided in Table 1. This table offers an overview of the processes and pre-treatments employed.

### 3.1. Evaluation Particle Size Distribution

Particle size distribution values of the stinging nettle dispersions used for protein extraction are given in Table 2. The xQ percentiles D10, D50 and D95 for high-pressure-treated stinging nettle dispersion show a significant difference from PEF as well as untreated samples. HPH-treated solutions demonstrate the effect of the process and the smallest values within the samples, as expected. The cell disruption effect of PEF can be seen in the slight difference from untreated samples. Since the particle size decrease can also take place due to cell disruption caused by freezing and thawing of the sample, the significant difference between PEF and untreated samples can not only be explained by the effect of PEF processing. The particle size distribution can be seen in Table 2.

When applied to microalgae like Chlorella vulgaris, PEF alone did not cause significant changes in particle size distribution. A significant reduction in particle size and cell fragmentation was observed only when PEF was combined with HPH [20].

### 3.2. Evaluation Protein Solubility

**Figure 5:**
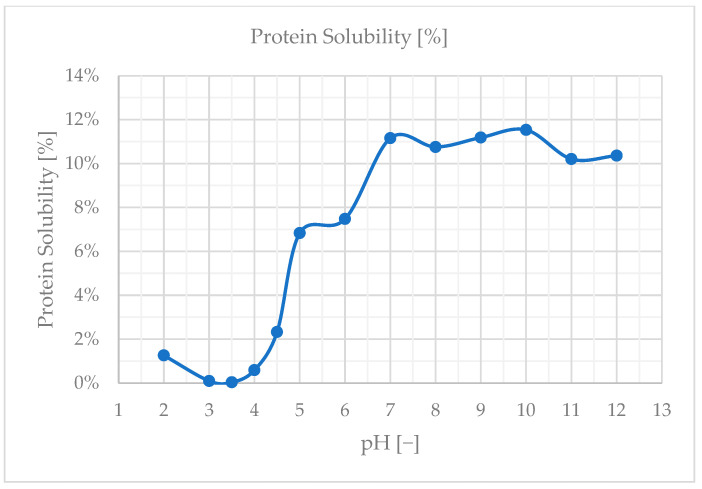
Protein solubility curve of SN5% solution.

The solubility characteristics of proteins are crucial for food processing. During various operations, proteins may encounter conditions that affect their solubility, which in turn influences the overall process. Therefore, it is essential to choose proteins that either stay soluble or precipitate under the desired processing conditions or the processing environment can be adjusted to match the solubility characteristics of the specific protein used. Stinging nettle leaf characterization has been studied by Bouazizi et al., 2022 as a novel source of protease [21]. This study delivers information about protease activity maximum at pH = 4. It can be expected that the maximum protease activity of the stinging nettle protein occurs when the proteins have minimum solubility. In the current study, protein solubility was unexpectedly low having a maximum solubility of only around 12%. The natural pH that settled for SN5% was observed at 8.3 across multiple batches. The protein solubility curve over a pH range of 2–12 can be seen in Figure 5. If we look at the minimum solubility area, we see no significant difference between pH values of 3 and 3.5, as well as 7 and 9. Based on the previous findings and current results, the isoelectric point of the sample is expected at a pH value of 3.5.

The determination of protein solubility revealed that the best solubility was achieved at a pH of 10, with the isoelectric point (PI) expected around pH 3.5. This suggests that the proteins in *Urtica dioica* leaves are more soluble in alkaline conditions, which could be leveraged to optimize extraction processes. According to Martin et al., 2019 [14], Rubisco proteins exhibit the lowest solubility at a pH of 5. However, they reported that protein solubility generally exceeds 80% following cell disruption, indicating that mechanical processing can significantly enhance solubility. In the study by Nynäs et al., 2021, the isoelectric precipitation (IEP) was noted at a pH of 4.5, which aligns with typical solubility patterns where proteins are least soluble near their isoelectric point [13]. Kronbauer et al., 2023 achieved a maximum solubility of 68%, highlighting the importance of solubility in improving protein yield [10]. This level of solubility is crucial for optimizing extraction efficiency and maximizing the recovery of protein from plant materials. Overall, understanding and manipulating solubility characteristics are key factors in enhancing protein extraction processes.

### 3.3. Evaluation Protein Content and Dry Mater

The total nitrogen (here used as equivalent to “protein”) content is crucial to understanding the suitability of a protein source as a dietary input for nutrition. Treatment methods and processing during extraction may alter the protein content by denaturation, by reacting with complex compounds as well as by breaking down the peptides due to alkaline or acid conditions. Since several technologies were applied, it was expected to see their intrinsic effects on the final nitrogen content. The values for all treatments can be seen in Table 3.

When comparing the pre-treatment methods HPH and PEF, it is obvious that HPH combined with IEP resulted in the highest protein content on dry matter, with the second-best treatment being PEF combined with IEP. Unfortunately, the UF and SO extraction processes did not yield satisfactory results. In the case of the UF extraction method, there was no significant difference between the pre-treatment methods used in this study. The protein content of *Urtica dioica* leaves in this study was found to be lower than the 32.3 ± 0.1% reported by Kronbauer et al., 2023 [10]. This discrepancy highlights potential variations due to factors such as plant variety, climate, soil conditions, and harvest time [22]. The protein extraction using ultrafiltration resulted in a lower protein content compared to the raw material. This outcome indicates that UF did not achieve the goal of concentrating the protein effectively, making it a less suitable method for enhancing protein yield from nettle leaves. Isoelectric precipitation (IEP) produced better results than UF but still did not achieve the desired concentration levels for a protein concentrate (>65%) or isolate (≥90%). This suggests that although IEP is more effective than UF, it is still inadequate for producing high-concentration protein products.

The second cycle of extraction utilizing the salting-in and salting-out effect yielded protein content similar to that of UF samples, which was also lower than the raw material. Although this approach did not significantly improve protein concentration, it demonstrated the potential for additional extraction cycles. However, given the modest yield improvement, pursuing further cycles may not be worthwhile from a practical standpoint.

Overall, these findings suggest that while certain extraction methods can enhance protein recovery from *Urtica dioica* leaves, achieving high-concentration protein products remains challenging with the techniques tested. Further optimization and exploration of alternative methods may be necessary to improve protein yield.

### 3.4. Evaluation Protein Yield

Another crucial parameter to be able to decide on the nutritive quality of a process or extraction product is protein yield. Maximizing protein yield is essential for cost-effective production as well, especially when it comes to new protein sources. Since the functionality of proteins depends on the quality and quantity of the extracted protein, higher yields guarantee that there is sufficient material to reach the desired functionality in final food products.

Previous studies on stinging nettle extraction reached protein yields in the range from 30 to 60% [10]. It should be noted that different measuring and protein extraction methods have been used in the mentioned study. In the current study, protein yields reached a max of approx. 12%. Despite low values of protein yield, the results provide further information about different processes. HPH in combination with IEP resulted in the highest yield. A possible improvement could be represented by using a lower cut-off value for the ultrafiltration process. One patent suggests a cut-off value between 30 and 50 kDa during concentration [23]. The results of protein yield and sample illustrations are shown in Table 4.

### 3.5. Evaluation Color Intensity

The color intensity of the samples is an important factor for the use of extracted proteins in final food matrices. Table 5 shows L* a* b* values of the different protein extracts produced by different methods, and their color profiles can be seen in Table 5.

Sample HPH_UF_SO has the highest L*-value at 66.82, followed by HPH_UF, PEF_UF, and PEF_IEP_SO. The last two samples also do not differ significantly from one another. This indicates that the protein extraction utilizing ultrafiltration resulted in a lighter color compared to the samples that were extracted using isoelectric precipitation. It also shows that a secondary extraction cycle with a 2M NaCl solution is especially beneficial for the lightness of the color, as PEF_IEP_SO is much lighter than the IEP samples. However, when considering protein yield, HPH_UF_SO and PEF_IEP_SO do not provide economical or ecological benefit for a two-staged extraction.

In this study, a perfectly white sample is described with the colorimetric values of L* = 100, a* = 0, b* = 0. This definition served as a reference point for evaluating the color traits of various samples. Among the samples tested, HPH_UF_SO emerged as the best in terms of color traits. However, when considering both yield and color traits, PEF_UF was identified as the most efficient sample. This dual consideration is crucial for applications where both visual appeal and material efficiency are important.

The a-values across all samples were generally moderate, yet they displayed significant differences among the samples. Notably, samples RAW and HPH_UF_SO exhibited a higher green hue, whereas other samples showed a predominance of red hue. This variation indicates distinct chromatic properties influenced by the processing methods applied to each sample.

Similar to a-values, b-values were significantly different across all samples. These values were more closely associated with the yellow spectrum rather than blue, suggesting a tendency towards warmer color tones in the processed samples.

According to [18], a color difference metric ΔE > 12 indicates that a distinctly different color is obtained, while values between 6 < ΔE < 12 suggest a strong difference. Findings in this study reveal that all processing methods applied resulted in at least a strong influence on the color composition of the extracted protein-rich fractions compared to the raw material. This underscores the substantial impact of processing techniques on altering the visual characteristics of these materials.

Chlorophyll is the most predominant secondary metabolite influencing the appearance of stinging nettle leaves. The amount of chlorophyll a and b and the total amount of raw material and extracted proteins is shown in Table 6.

The RAW sample shows the highest chlorophyll content across all categories. In contrast, the PEF_UF sample shows the lowest chlorophyll levels. Significant differences are observed among the samples, as indicated by differing lowercase letters next to the values.

Chlorophyll a, chlorophyll b, and their total amount decreased for all extraction methods, with the raw material maintaining the highest chlorophyll amount. The ultrafiltration process is more effective than the IEP process in reducing chlorophyll content, regardless of whether cell disruption was achieved through HPH or PEF. An exception to this is the HPH_UF_SO sample, where the chlorophyll levels of an ultrafiltration method are closer to those of an IEP extraction. This suggests that during the secondary extraction process, more proteins bound to chlorophyll are extracted. These proteins were effectively removed in the HPH_UF sample, as intended by the method [14]. The Chlorophyll a, b, a + b amount per gram of powder is summarized in Table 7.

This study demonstrates that all extraction processes significantly reduced the chlorophyll content when processing stinging nettle leaves. When comparing the chlorophyll values of *Phaseolus vulgaris* leaves, which range from 1340 to 2440 µg/g [24], it is evident that the values in *Urtica dioica* leaves are quite high, even within the category of leaves, at 4781.41 µg/g. The greatest success was observed with the PEF_UF method, which reduced Chlorophyll a from 3766.28 µg/g in raw leaves to 9.96 µg/g and chlorophyll b from 1015.13 µg/g to 5.11 µg/g, achieving a total chlorophyll reduction to 15.07 µg/g compared to the initial 4781.41 µg/g. The results from the HPH_UF_SO sample do not support the assumption that a secondary extraction cycle is beneficial in terms of decreasing the color intensity.

### 3.6. Comparison of Extraction Efficiency and Color Reduction

This study investigates various cell disruption methods and extraction techniques to optimize protein yield and chlorophyll reduction from stinging nettle leaves. The methods compared include HPH and PEF for cell disruption, and IEP, UF, and SO for extraction. These results can be seen in Table 8.

The results indicate that the combination of HPH + IEP yields the highest protein content on a dry matter basis, making it the most effective method for maximizing protein recovery from stinging nettle leaves in this study. In contrast, Ece Goktayoglu et al. (2023) investigated protein extraction from sugar beet leaves, reporting yields of 29.91% for isoelectric point precipitation, 16.65% for heat coagulation, and 35.81% for ammonium sulfate precipitation [9]. This study explored various precipitation methods to extract proteins from agricultural waste, highlighting the potential of sugar beet leaves as a protein source. Meanwhile, Nynäs et al. (2021) examined protein fractionation from different green biomass types, including sugar beet leaves. Their study found that mangold leaves resulted in the highest nitrogen yield of 1.9% in the white protein fraction [13]. Both studies illustrate the differences in protein yields based on plant source and extraction method. In comparison, this study achieves a moderate yield from nettle leaves compared to other methods applied to sugar beet and mangold leaves.

When considering chlorophyll reduction, PEF + UF proved to be the most effective method, achieving the lowest levels of residual chlorophyll. The study highlights that while HPH combined with IEP is superior in terms of protein yield, PEF combined with UF is more efficient in reducing chlorophyll content, which is crucial for applications where visual appeal is important. The findings suggest that combining mechanical cell disruption with chemical precipitation or filtration can enhance both the protein extraction efficiency and color quality of the final product. Further optimization could focus on balancing these two aspects to achieve a higher concentration of protein products. However, the overall protein yield was relatively low in this study, with the highest being 11.60% for HPH combined with IEP. In contrast, sugar beet leaves have been shown to achieve higher protein yields using various precipitation methods, including isoelectric point precipitation and high-pressure-assisted techniques. These methods have been effective in extracting proteins with yields above 80% for certain proteins, indicating a more efficient extraction process compared to stinging nettle. Overall, while both stinging nettle and sugar beet leaves present viable sources of plant-based proteins, the extraction efficiency and yield vary significantly depending on the method used. The choice of extraction technique should consider the desired protein content and yield, as well as the specific application of the extracted proteins. The study by Alattar et al. (2022) demonstrates that Chlorella vulgaris isolates have a significantly higher chlorophyll content, measuring 18,600 µg/g, compared to stinging nettle isolates [25]. In contrast, raw stinging nettle leaves contain 4781.41 µg/g of chlorophyll, which decreases to 1817.74 µg/g when extracted using HPH combined with IEP. The PEF combined with the UF method further reduces the chlorophyll content in stinging nettle to just 15.07 µg/g. This comparison underscores that Chlorella isolates retain much higher chlorophyll levels than stinging nettle isolates, regardless of the extraction method. Notably, protein extracts from stinging nettle are not widely utilized, and comparing them with established values is challenging due to limited research.

## 4. Conclusions

The study investigates the impact of various extraction technologies on protein and chlorophyll yield from stinging nettle, highlighting the effectiveness of different methods in optimizing protein recovery and reducing color intensity. Among the evaluated techniques, pulsed electric fields and ultrafiltration were particularly effective in decreasing color intensity, with pulsed electric fields achieving the most significant reduction in chlorophyll content, making it promising for applications where visual appeal is crucial. Isoelectric precipitation demonstrated the highest protein yield, outperforming other methods like ultrafiltration and a two-stage extraction utilizing salting-in and salting-out, indicating its potential as a preferred technique for maximizing protein recovery. The study also emphasizes that protein solubility is highly variable and dependent on factors such as batch differences and harvest conditions, making it critical to optimize for increasing protein yield. High-pressure homogenization, when combined with isoelectric precipitation, resulted in the highest protein yield among the tested methods, suggesting that combining mechanical cell disruption with chemical precipitation can enhance protein extraction efficiency. Despite these promising results, further optimization is necessary to improve the extraction processes. Evaluating the nutritional value and sensory attributes of the extracted proteins is essential for their successful application in food products. Future research should focus on refining these techniques to achieve higher concentration protein products while maintaining desirable color and sensory properties. This study provides a foundation for utilizing stinging nettle as a sustainable protein source, paving the way for its broader use in food fortification and functional ingredient development.

## Figures and Tables

**Table 1:** Nomenclature for samples and processes.

Abbreviation	Meaning
RAW	Untreated raw material
HPH	Cell disruption via HPH
PEF	Cell disruption via PEF
IEP	Extraction via IEP
UF	Extraction via UF
SO	Extraction via salting-out

**Table 2:** Particle size distribution of untreated, high-pressure homogenization and pulsed electric field-treated stinging nettle dispersion.

Sample	D10 [µm]	D50 [µm]	D90 [µm]
SN5%_RAW	18.1 ± 0.26 a	87.17 ± 0.76 d	181.6 ± 5.8 f
SN5%_HPH	5.56 ± 0.03 b	41.66 ± 0.33 e	134.6 ± 0.9 g
SN5%_PEF	16.54 ± 0.06 c	88.34 ± 0.47 d	189.3 ± 1.1 f

Values with the same letter indicate no significant difference at α = 0.05.

**Table 3:** Protein content, dry matter and protein content based on dry matter for different treatment methods.

Sample [%]	Protein Content [%]	Dry Matter [%]	Protein Content Based on Dry Matter [%]
RAW	22.85 ± 0.69 c	91.74 ± 0.32 b	24.91
HPH_IEP	44.42 ± 0.27 a	90.47 ± 0.30 c	49,09
HPH_UF	17.29 ± 0.54 d	88.92 ± 0.45 d	19.45
HPH_UF_SO	13.71 ± 0.47 f	92.50 ± 0.45 b	14.82
PEF_IEP	39.23 ± 0.11 b	91.47 ± 0.41 b,c	42.89
PEF_IEP_SO	15.88 ± 0.15 e	94.32 ± 0.20 a	16.84
PEF_UF	17.33 ± 0.27 d	86.12 ± 0.63 e	20.12

Values with the same letter indicate no significant difference at α = 0.05.

**Table 4:** Protein yield [%] and color profile of extracted samples with different methods.

**RAW**	**HPH_IEP**	**HPH_UF**	**HPH_UF_SO**
100%	11.60%	2.27%	0.27%
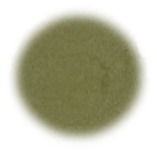	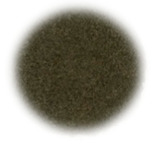	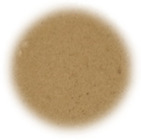	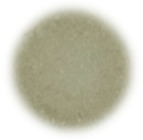
	**PEF_IEP**	**PEF_UF**	**PEF_IEP_SO**
	2.96%	2.68%	0.58%
	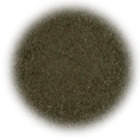	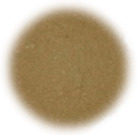	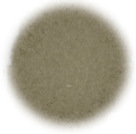

**Table 5:** L* a* b* values of samples (−a = geen, +a = red, −b = blue, +b = yellow).

Sample	L*	a*	b*	ΔΕ
RAW	47.10 ± 0.01 d	−2.99 ± 0.03 g	22.81 ± 0.04 c	0
HPH_IEP	25.20 ± 0.01 f	3.44 ± 0.01 c	11.78 ± 0.04 f	25.35
HPH_UF	56.91 ± 0.07 b	8.38 ± 0.03 a	27.74 ± 0.02 a	15.81
HPH_UF_SO	66.82 ± 0.49 a	−2.64 ± 0.01 f	16.10 ± 0.19 d	20.83
PEF_IEP	30.51 ± 0.13 e	2.27 ± 0.10 d	11.41 ± 0.03 g	20.81
PEF_UF	53.39 ± 0.13 c	7.84 ± 0.02 b	26.85 ± 0.05 b	13.16
PEF_IEP_SO	52.87 ± 0.38 c	0.95 ± 0.01 e	14.01 ± 0.04 e	11.24

Values with the same letter indicate no significant difference at α = 0.05.

**Table 6:** Chlorophyll a, chlorophyll b and total chlorophyll values of samples.

Sample	Chl a [μg]	Chl b [μg]	Chl a + b [μg]
RAW	69.10 ± 0.11 a	18.63 ± 0.06 a	87.73 ± 0.05 a
HPH_IEP	32.74 ± 0.06 b	1.63 ± 0.04 d	34.37 ± 0.03 b
HPH_UF	1.48 ± 0.02 c	0.24 ± 0.02 e,f	1.71 ± 0.03 c
HPH_UF_SO	32.52 ± 0.05 d	4.88 ± 0.02 b	37.41 ± 0.03 d
PEF_IEP	5.04 ± 0.02 e	2.13± 0.10 c	7.17 ± 0.10 e
PEF_UF	0.18 ± 0.04 f	0.09 ± 0.07 f	0.28 ± 0.04 f
PEF_IEP_SO	3.13 ± 0.06 g	0.26 ± 0.06 e	3.39 ± 0.02 g

Values with the same letter indicate no significant difference at α = 0.05.

**Table 7:** Chlorophyll a, b, a + b amount per gram of powder.

Sample	Chl a [μg/g]	Chl b [μg/g]	Chl a + b [μg/g]
RAW	3766.28 ± 5.83 a	1015.13 ± 3.26 a	4781.41 ± 2.76 a
HPH_IEP	1731.55 ± 3.31 b	86.18 ± 2.36 d	1817.74 ± 1.78 c
HPH_UF	82.97 ± 0.64 f	13.24 ± 1.18 e	96.21 ± 1.83 f
HPH_UF_SO	1706.86 ± 2.62 c	256.16 ± 1.12 b	1963.02 ± 1.77 b
PEF_IEP	268.883 ± 1.21 d	113.38 ± 5.36 c	382.26 ± 5.22 d
PEF_UF	9.96 ± 2.01 g	5.11 ± 3.95 e	15.07 ± 2.16 g
PEF_IEP_SO	162.64 ± 3.06 e	13.71 ± 3.24 e	176.35 ± 1.28 e

Values with the same letter indicate no significant difference at α = 0.05.

**Table 8:** Comparison of extraction efficiency and color reduction.

Method	Protein Yield [%]	Chlorophyll Reduction [µg/g]	Comments
RAW	100	0	Untreated raw material with highest chlorophyll content.
HPH_IEP	11.60	2963.67	Highest protein yield among methods tested, significant chlorophyll reduction.
HPH_UF	2.27	4685.20	Lower protein yield, effective chlorophyll reduction.
HPH_UF_SO	0.27	2818.39	Minimal protein yield, moderate chlorophyll reduction.
PEF_IEP	2.96	4399.15	Moderate protein yield, effective chlorophyll reduction.
PEF_UF	2.68	4766.34	Low protein yield, most effective chlorophyll reduction.
PEF_IEP_SO	0.58	4605.06	Low protein yield, effective chlorophyll reduction.

Values were calculated by the average values.

## Data Availability

The original contributions presented in the study are included in the article, further inquiries can be directed to the corresponding author.
